# DrImpute: imputing dropout events in single cell RNA sequencing data

**DOI:** 10.1186/s12859-018-2226-y

**Published:** 2018-06-08

**Authors:** Wuming Gong, Il-Youp Kwak, Pruthvi Pota, Naoko Koyano-Nakagawa, Daniel J. Garry

**Affiliations:** Lillehei Heart Institute, University of Minnesota, 2231 6th St S.E, 4-165 CCRB, Minneapolis, MN 55114 USA

**Keywords:** Single cell RNA sequencing data, Dropout events, Imputation, Next generation sequencing

## Abstract

**Background:**

The single cell RNA sequencing (scRNA-seq) technique begin a new era by allowing the observation of gene expression at the single cell level. However, there is also a large amount of technical and biological noise. Because of the low number of RNA transcriptomes and the stochastic nature of the gene expression pattern, there is a high chance of missing nonzero entries as zero, which are called dropout events.

**Results:**

We develop DrImpute to impute dropout events in scRNA-seq data. We show that DrImpute has significantly better performance on the separation of the dropout zeros from true zeros than existing imputation algorithms. We also demonstrate that DrImpute can significantly improve the performance of existing tools for clustering, visualization and lineage reconstruction of nine published scRNA-seq datasets.

**Conclusions:**

DrImpute can serve as a very useful addition to the currently existing statistical tools for single cell RNA-seq analysis. DrImpute is implemented in R and is available at https://github.com/gongx030/DrImpute.

**Electronic supplementary material:**

The online version of this article (10.1186/s12859-018-2226-y) contains supplementary material, which is available to authorized users.

## Background

DNA sequencing technology and next generation sequencing approaches for high-throughput RNA sequencing are experiencing tremendous growth. Bulk RNA sequencing (bulk RNA-seq) technology performs high throughput sequencing of RNA isolated from millions of cells, which implies that the resulting expression value for each gene is the average expression value of a large population of input cells [1, 2]. Thus, bulk RNA-seq is suitable for revealing a global view of averaged gene expression levels. However, the bulk RNA-seq method is not capable of quantifying the RNA contents of a limited number of cells and yields bias the results when samples consist of heterogeneous cell populations. For example, bulk RNA-seq is unable to accurately reveal the transcriptome of the cells from the early embryonic developmental stage where there exists multiple lineages with a relatively limited number of cells. Recently, scRNA-seq was developed to enable a wide variety of transcriptomic analyses at the single cell level [3–5]. The major areas in scRNA-seq research include characterization of the global expression profiles of rare cell types, the discovery of novel cell populations, and the reconstruction of cellular developmental trajectories [6–9]. Accordingly, many statistical methods have been developed for the clustering of cell populations, the visualization of cell-wise hierarchical relationships, and the prediction of lineage trajectories [9–22].

However, scRNA-seq has a relatively higher noise level than bulk RNA-seq especially due to so-called dropout events [10–14]. The observed zeros in the gene-cell expression matrix of the scRNA-seq datasets consist of *true zeros*, where the genes are not expressed at all, and the *dropout zeros* are due to the so-called dropout events [10]. Dropout events are special types of missing values (a missing value is an instance wherein no data are present for the variable), caused both by low RNA input in the sequencing experiments and by the stochastic nature of the gene expression pattern at the single cell level. However, most statistical tools developed for scRNA-seq analysis do not explicitly address these dropout events [2]. We hypothesize that imputing the missing expression values caused by the dropout events will improve the performance of cell clustering, data visualization, and lineage reconstruction.

The gene expression data from bulk RNA-seq (or microarrays) are also challenged from a missing value problem [15]. Various statistical methods have been proposed to estimate the missing values in the data [16, 17]. These missing value imputation methods can be categorized as five general strategies, as follows: (1) *mean imputation* estimates missing entries by averaging gene-level or cell-level expression levels [16–19]; (2) *hot deck imputation* predicts missing values from similar entries using a similarity metric among genes (KNNImpute [17]); (3) *model based imputation* employs statistical modeling to estimate missing values (GMCimpute [16]); (4) *multiple imputation* methods predict missing entries multiple times and the combination of the results to produce final imputation (SEQimpute [18]); and (5) *cold deck imputation* uses side information such as gene ontology to facilitate the imputation process (GOkNN, GOLLS [19]).

However, the imputation methods developed for bulk RNA-seq data may not be directly applicable to scRNA-seq data. First, much larger cell-level variability exists in scRNA-seq, because scRNA-seq has cell-level records for gene expression; on the other hand, bulk RNA-seq data have the averaged gene expression of the population of cells. Second, dropout events in scRNA-seq are not exactly missing values; dropout events have zero expression, and they are mixed with real zeros. In addition, the proportion of missing values in bulk RNA-seq data is much smaller. Therefore, a dropout imputation model for scRNA-seq is needed.

There are a few previous studies for imputing dropout events [20–24]. BISCUIT iteratively normalizes, imputes, and clusters cells using the Dirichlet process mixture model [22]. Zhu et al. proposed a unified statistical framework for both single cell and bulk RNA-seq data [20]. In their method, the bulk and single cell RNA-seq data are linked together by a latent profile matrix representing unknown cell types. The bulk RNA-seq datasets are modeled as a proportional mixture of the profile matrix and the scRNA-seq datasets are sampled from the profile matrix, considering the dropout events. The scImpute infers dropout events with high dropout probability and only perform imputation on these values [23]. MAGIC imputes the missing values by considering similar cells based on heat diffusion, though MAGIC would alter all gene expression levels including those non-zero values [24]. However, none of these studies have systematically demonstrated how imputing dropout events could improve the current statistical methods that do not account for dropout events.

In the present study, we designed a simple, fast hot deck imputation approach, called DrImpute, for estimating dropout events in scRNA-seq data. DrImpute first identifies similar cells based on clustering, and imputation is performed by averaging the expression values from similar cells. To achieve robust estimations, the imputation is performed multiple times using different cell clustering results followed by averaging multiple estimations for final imputation. We demonstrated using nine published scRNA-seq datasets that imputing the dropout entries significantly improved the performance of existing tools, including pcaReduce [25], SC3 [26], t-SNE [27], PCA, Monocle [28], and TSCAN [29], with regards to cell clustering, visualization, and lineage reconstruction. Moreover, DrImpute also performed better than CIDR [30], ZIFA [31], scImpute [23] and MAGIC [24] in accounting for dropout events in scRNA-seq data.

## Results

### scRNA-seq datasets

In this study, we used nine published scRNA-seq datasets to comprehensively examine the performance of DrImpute on imputing the zeros in the scRNA-seq data and whether the imputation would improve the performance of existing analysis tools. Table 1 summarized these nine scRNA-seq datasets. We grouped these datasets into three levels (gold, silver and bronze) based on the supporting evidence of the reported cell labels [26]. The “gold standard” dataset included: Pollen [8], Blakeley [32] and Zheng [33] datasets, where the cell labels were defined based on experimental conditions or cell lines. Thus, the cells within each condition were relatively homogenous. The “silver standard” datasets included: Usoskin [34] and Hrvatin [35] datasets, where the cell labels were computationally derived and assigned based on the authors’ knowledge of the underlying biology. The cell labels of the remaining four “bronze standard” datasets (Deng [6], Treutlein [36], Scialdone [37] and Petropoulos [38]) were developmental stages (time labels). Although the single cell populations from different time points usually have distinct expression patterns and biological characteristics, the time labels per se were unable to separate the distinct populations within each time point. Thus, for the “bronze standard” datasets, the cell labels (time labels) may understate the existing cell populations.

**Table 1:** The scRNA-seq datasets used for comparing the performance of different tools

Dataset	k	# cells	Standard	Cell Label Definition	UMI	Ref
Pollen	11	301	gold	human cell lines	No	[8]
Deng	10	286	bronze	Stages of mouse preimplantation development	No	[6]
Usoskin	4	622	silver	Clusters of mouse lumbar DRG (dorsal root ganglion)	No	[35]
Blakeley	3	30	gold	Human pluripotent epiblast cells, extraembryonic trophectoderm cells and primitive endoderm cells	No	[32]
Treutlein	8	405	bronze	Cell populations from direct reprogramming from fibroblast to neuron (MEF, day 2, 5, and 22)	No	[36]
Zheng	10	94,655	gold	Cell populations from human immune system	Yes	[33]
Hrvatin	8	48,266	silver	Cell populations from mouse visual cortex	Yes	[34]
Scialdone	4	1205	bronze	Stages of mouse mesodermal development	No	[37]
Petropoulos	5	1529	bronze	Stages of human preimplantation development	No	[38]

*k* represents the number of cell clusters reported in the original study. Datasets were grouped into three levels (gold, silver and bronze standards) based on the supporting evidence of the reported cell labels

### DrImpute has significantly better performance on the separation of the dropout zeros from true zeros

**Fig. 1:**
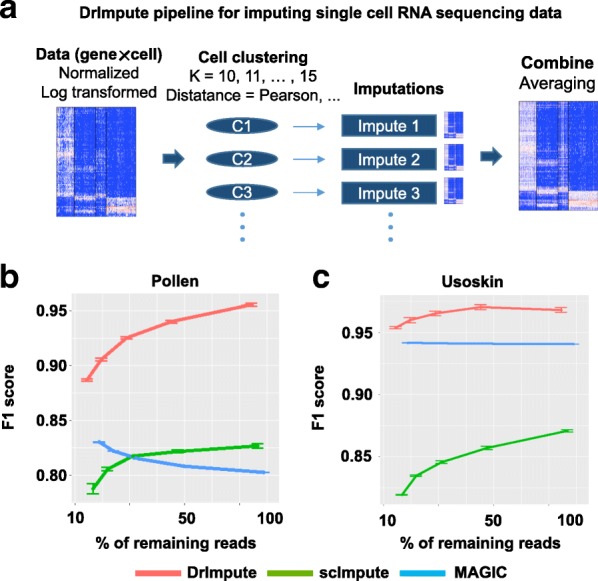
DrImpute has significantly better performance on discriminating dropout zeros from true zeros than existing methods. (**a**) Overview of DrImpute pipeline: (1) data cleansing, normalization, and log transformation; (2) calculating the distance matrix among cells; (3) imputing the dropout entries based on the clustering results; and (4) averaging all imputation results to determine the final imputation. **b**-**c** Three scRNA-seq imputation algorithms DrImpute, scImpute and MAGIC were used to discriminating the dropout zeros from the true zeros in the simulation studies. The full scRNA-seq datasets from (**b**) Pollen et al. and (**c**) Usoskin et al. were down-sampled at 10, 15%, 25, 40 and 63% of the total number of reads. The discriminative performance was measured by F1 score (the harmonic mean of precision and recall)

Figure 1 summarized the general computational framework of DrImpute. First, the cell-cell distance matrix was computed using Spearman and Pearson correlations, followed by the cell-wise clustering based upon the distance matrix over a range of expected number of clusters *k* (*k* ranging from 10 to 15 by default). For each combination of distance metric (Spearman or Pearson) and *k*, we estimated the zero values in the input gene-cell matrix. The averaged estimation over all combinations were taken as the final imputed values (see Methods).

We first investigated the performance of DrImpute on discriminating true zeros and dropout zeros in the scRNA-seq data matrix using a down-sampling based simulation method. We defined the *true zeros* as the genes where expression levels are consistently zero across all cells belonging to one cell cluster (Additional file 1: Figure S2). To generate the *dropout zero*, we randomly down-sampled the raw sequencing reads to 10, 15, 25, 40 and 63% (10^− 1^, 10^–0.8^, 10^–0.6^, 10^–0.4^ and 10^–0.2^) of the total number of reads, mapped the sampled reads onto the genome and computed the corresponding gene-cell read count matrices. We defined *dropout zero* as the genes where expression levels were zero in the down-sampled datasets, but were positive in the full dataset.

Then, we utilized DrImpute, along with two other published scRNA-seq imputation tools scImpute and MAGIC to impute zero events in the down-sampled dataset. The imputed zero events could therefore be grouped into four situations: (1) true positive (TP, imputed dropout zeros), (2) true negative (TN, non-imputed true zeros), (3) false positive (FP, imputed true zeros) and (4) false negative (FN, non-imputed dropout zeros). The F1 score (the harmonic mean of precision and recall) was used to evaluate the imputation performance of each method on down-sampled datasets. We found that DrImpute had consistently better performance of discriminating the true zeros and the dropout zeros at various down-sampling ratio on both Pollen and Usoskin datasets (Fig. 1b and c).

### DrImpute significantly improved the performance of existing tools for cell type identification

Discovering distinct cell types from a heterogeneous cell population (cell clustering) is one of the most important applications of scRNA-seq. Several methods, such as pcaReduce [25], SC3 [26], and t-SNE followed by k-means (t-SNE/kms), have been developed and utilized for clustering scRNA-seq data. However, these methods did not explicitly address the dropout events or the missing values existing in the scRNA-seq data. We hypothesized that (1) preprocessing the scRNA-seq data by imputing the dropout events via DrImpute will improve the accuracy of these clustering methods and (2) the performance of the existing tools combined with DrImpute will perform better than existing scRNA-seq imputation tools such as CIDR [30], scImpute [23] and MAGIC [24] in addressing dropout events.

**Fig. 2:**
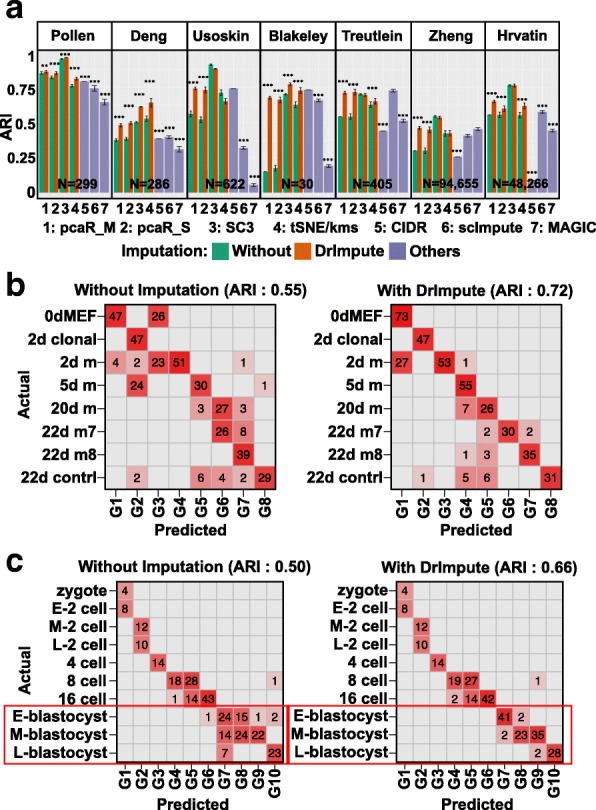
DrImpute significantly improved the performance of the existing tools for cell type identification. (**a**) The average adjusted Rand index (ARI) of 100 repeated runs of pcaR_M (pcaReduce with the M option), pcaR_S (pcaReduce with the S option), SC3, t-SNE/kms (t-SNE followed by k-means), CIDR, scImpute and MAGIC, on seven scRNA-seq datasets. For Zheng and Hrvatin datasets, 1000 cells were randomly sampled from the full datasets and used for the clustering analysis for each method. Black interval represents one plus or minus standard error of the category. Wilcoxon rank sum test was utilized to compare the ARIs from different tools (∗∗: 0.01 ≤ *p* value < 0.001, ∗∗∗ *p* value < 0.001). **b**-**c** The confusion matrix for (**b**) iN reprograming using pcaReduce (option S) and (**c**) mouse preimplantation embryo using t-SNE followed by k-means. Y axis represents ground truth cluster groups reported in the original study and X axis represents predicted groups. Left and right panels, respectively, represent the confusion matrix according to the clustering results without and with preprocessing the scRNA-seq data using DrImpute. The ARI was computed between the original and predicted cell groups

First, we evaluated whether imputing the dropout events using DrImpute before applying pcaReduce, SC3, and t-SNE would improve the accuracy of cell type identification. We compared the clustering performance of these methods with and without imputing dropout events by DrImpute, on seven published scRNA-seq datasets. Using the cell types reported in the original publications as the ground truth and the Adjusted Rand Index (ARI) as the performance metric, we observed that preprocessing the scRNA-seq datasets with DrImpute significantly improved the clustering performance of pcaReduce with the M and S options (pcaR_M: merging based on largest probability; pcaR_S: sampling based merging) on all seven tested datasets; improved the performance of t-SNE followed by k-means (t-SNE/kms) on five datasets; and improved the performance of SC3 on three datasets (Fig. 2a). Second, we also found that combining DrImpute with t-SNE/kms showed significantly better clustering performance than CIDR on five of seven datasets, scImpute followed by t-SNE/kms on five of seven datasets, MAGIC followed by t-SNE/kms on six of seven datasets (Fig. 2a).

Figure 2b shows a confusion matrix of the ground truth cell labels and cell clusters predicted by pcaReduce (option S) on the scRNA-seq dataset of induced neuronal (iN) reprograming, without (left panel) and with (right panel) imputing the dropout events using DrImpute. We observed a clearer diagonal pattern in the confusion matrix with the imputation, as supported by an improvement of ARI, from 0.55 to 0.72. As another example, Fig. 2c showed the confusion matrix of the ground truth labels and cell clusters predicted by t-SNE/kms on a dataset of mouse preimplantation embryos. We found that imputing the dropout events facilitated t-SNE/kms more accurately to cluster the cells from the blastocyst stages, as evidenced by an increase in mean ARI from 0.50 to 0.66.

We further assessed whether preprocessing scRNA-seq by imputing dropout events would produce more consistent clustering results. We evaluated the robustness of the clustering results with and without imputing the dropout events using DrImpute. We hypothesized that preprocessing the scRNA-seq with DrImpute would facilitate the clustering methods to detect more robust and consistent subpopulations. For each dataset, we randomly sampled 100 genes (gene level down-sampling), or one-third of the total cells (cell level down-sampling), and we clustered the cells using each of the clustering methods with and without preprocessing the down-sampled dataset using DrImpute. This process was repeated 100 times, and we compared how consistent the clustering results were after down-sampling the genes or cells as measured by cross ARI (see Methods). For both the gene and cell down-sampling experiments, we found that preprocessing of the scRNA-seq datasets with DrImpute significantly improved the robustness of the cell type identification of SC3, t-SNE/kms, and pcaR_M/pcaR_S on 80% of the tested cases (Additional file 1: Figure S5a and b).

In summary, these results suggested that in 55 out of 66 (83%) tested cases, preprocessing the scRNA-seq datasets by imputing the dropout events using DrImpute significantly improved the accuracy or the robustness of clustering methods that did not specifically address dropout events. Compared with other scRNA-seq imputation tools such as scImpute, CIDR and MAGIC, DrImpute combined with t-SNE/kms had improved clustering performance on 16 of 21 (76.2%) tested cases.

### DrImpute significantly improved the performance of PCA and t-SNE in visualizing scRNA-seq data

Principal component analysis (PCA) and t-SNE are among the most popular methods for visualizing scRNA-seq in a two- (2D) or three-dimensional (3D) space. However, neither PCA nor t-SNE explicitly addressed dropout events. Zero Inflated Factor Analysis (ZIFA) was the first specific tool designed for factorizing and visualizing scRNA-seq data [31], followed by a few recent methods [39, 40]. We hypothesized that with the preprocessing of scRNA-seq data by imputing the dropout events using DrImpute, the generic dimension reduction methods (PCA and t-SNE) would generate better factorization or visualization results than without imputation.

To evaluate the accuracy of the dimension reduction in 2D space, we first estimated how discriminatively the cells from one population (using the class label reported in the original publication) separated from other populations in 2D space. For each dimension reduction result, we used the 2D coordinates of 90% of cells as the feature to train a linear support vector machine (SVM) classifier, and we predicted the class label for the remaining 10% of the cells. The above process was repeated ten times, and the overall prediction accuracy (10-fold cross validation accuracy) was used to quantitatively measure the separation of different populations in 2D space.

**Fig. 3:**
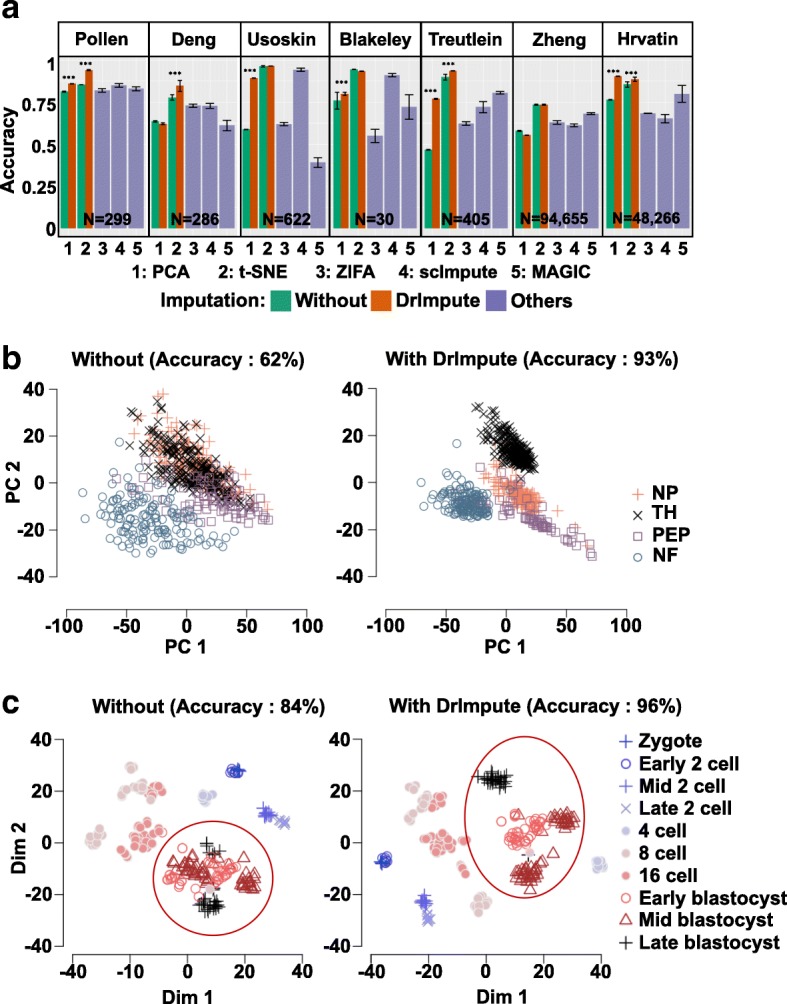
DrImpute significantly improved the performance of PCA and t-SNE in visualizing scRNA-seq data. **a** The barplots of average accuracy of separating the cell subpopulations in 2D space. For Zheng and Hrvatin datasets, 1000 cells were randomly sampled from the full datasets and used for the clustering analysis for each method. Black interval represents one plus or minus standard error of the category. Wilcoxon rank sum test was utilized to compare the accuracy from different tools (***p value <0.001). **b** Visualization of four groups of mouse neural single cells (NP, TH, PEP, and NF) using PCA. Left and right panels, respectively, show the 2D visualization of single cells without and with preprocessing the scRNA-seq data using DrImpute. **c** Visualization of mouse preimplantation embryo using t-SNE. Left and right panels, respectively, show the 2D visualization of single cells without and with preprocessing the scRNA-seq data using DrImpute. The classification accuracy was computed by using the 2D coordinates of each dimension reduction results

We compared the performance of PCA and t-SNE with and without DrImpute preprocessing as well as ZIFA and t-SNE with scImpute on seven published scRNA-seq datasets. We observed significant improvements in PCA or t-SNE with DrImpute on 9 of 14 (64.3%) tested cases (Fig. 3a). Moreover, using three datasets (Pollen, Usoskin and Treutlein) where ZIFA had better separation than PCA, preprocessing the data with imputation employing DrImpute helped PCA achieve significantly better performance than ZIFA in separating the cell populations (Fig. 3a). Comparison with imputing data with scImpute and MAGIC followed by t-SNE, DrImpute showed significantly better visualization performance on 12 of 14 (85.7%) tested cases (Fig. 3a).

Figure 3b depicted the cell expression profiles of four types of neurons (non-peptidergic nociceptors (NP), tyrosine hydroxylase containing (TH), peptidergic nociceptors (PEP), and neurofilament containing (NF)) in mice using PCA without (left) and with (right) imputing the dropout events using DrImpute. Without imputing the dropout events with DrImpute, the NP, TH, and PEP groups were visually indistinguishable in the 2D space. However, after applying DrImpute, all four groups were visually separated, as demonstrated by an accuracy increase from 62 to 93%. Fig. 3c showed the cell expression profiles of mouse preimplantation embryos using t-SNE. As seen in the red circled area, the stages of early, mid, and late blastocyst were more clearly distinguished after preprocessing the scRNA-seq data with DrImpute, as supported by an accuracy increase from 84 to 96%.

In summary, we found that preprocessing the scRNA-seq datasets by imputing the dropout events using DrImpute significantly improved the accuracy of visualization. The generic dimension reduction methods (PCA and t-SNE) on imputed datasets using DrImpute also performed significantly better than ZIFA, which was specifically designed for scRNA-seq data considering dropout events.

### DrImpute significantly improved the performance of monocle and TSCAN in lineage reconstruction

The third common task for single cell RNA-seq analysis is to reconstruct the lineage trajectories and infer the differentiated and progenitor states of the single cells. For example, Monocle [28] and TSCAN [29] were designed to infer pseudotime from the biological cellular process. However, neither method accounted for dropout events. We hypothesized that inferring the pseudotime on scRNA-seq data preprocessed using DrImpute could improve the accuracy of pseudotime ordering.

We compared the performance of pseudotime inference with and without imputing the dropout events on three published temporal scRNA-seq datasets, mouse preimplantation embryonic development data (Deng [6]), human preimplantation embryonic development data (Petropoulos [38]), and mouse early mesodermal development data (Scialdone [37]). The Deng dataset included the single cells from ten early mouse developmental stages from zygote, 2−/4−/8−/16- cell stages to blastocyst. The Petropoulos dataset included the single cells from five stages of human preimplantation embryonic development from developmental day (E) 3 to day 7. The Scialdone dataset included the single cells from four stages of early mesodermal development at E6.5, E7.0, E7.5 and E7.75 in the mouse. It should be noted that although the cells within each of the time points may not be homogenous, the time labels could be used to represent the overall developmental trajectory, and to evaluate the performance of pseudotime inference algorithms [41–45]. Thus, we used the reported time labels as the ground truth and evaluated the performance of pseudotime inference by comparing the time labels and pseudotime. The consistency between the time labels and pseudotime ordering was measured by the Pseudo-temporal Ordering Score (POS) and Kendall’s rank correlation score.

**Fig. 4:**
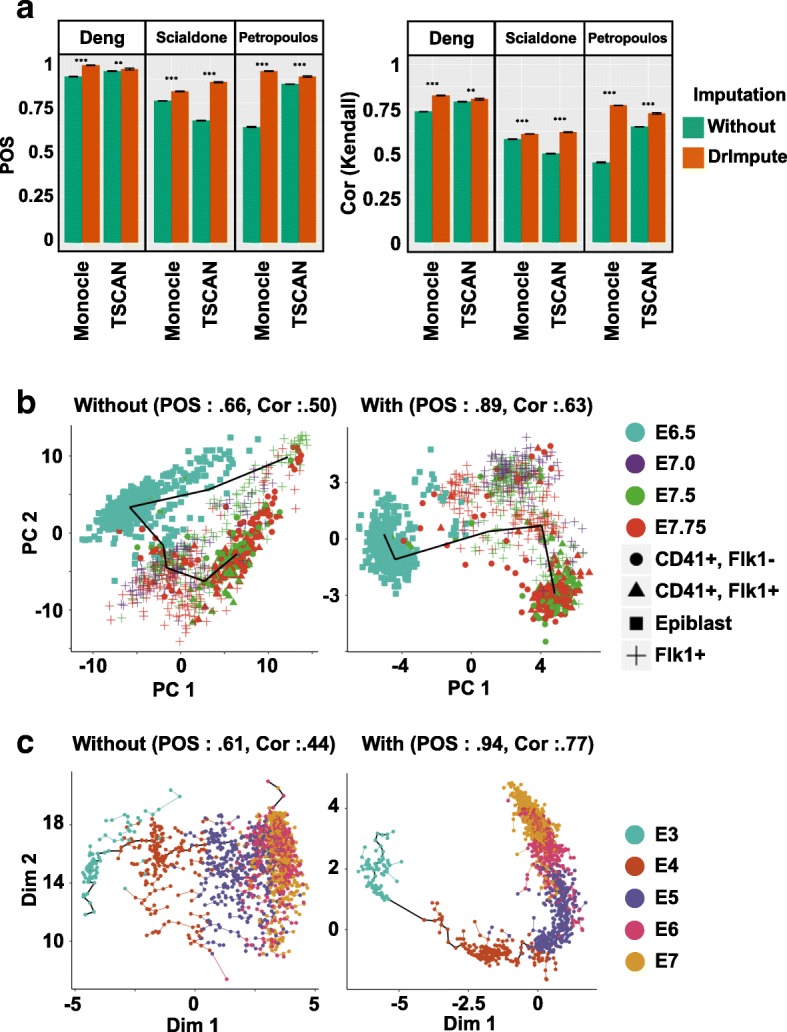
DrImpute greatly improved the performance of Monocle and TSCAN in lineage reconstruction. **a** The barplots of averaged Kendall’s rank correlation score and POS of 100 repeated runs of Monocle and TSCAN on three time series scRNA-seq datasets. Blue interval represents one plus or minus standard deviation of the category. Black interval represents one plus or minus standard error of the category. Both TSCAN and Monocle are deterministic with 0 variation before imputation. Wilcoxon rank sum test was utilized to compare Kendall’s rank correlation score and POS from different tools (***p value <0.001). **b** Visualization of lineage reconstruction of mouse early mesoderm using TSCAN. The left and right panels, respectively, show the results of lineage reconstruction by TSCAN using the un-imputed scRNA-seq data or data preprocessed using DrImpute. The “Flk1+” cell population represents mesodermal cells. “Epiblast” is the outermost layer of an embryo before it differentiates into ectoderm and mesoderm around mouse developmental day (E) 6.5. The CD41+/Flk1- cell population represents the mature hematopoietic lineage, and CD41+/Flk1+ cell population represents an early hematopoietic lineage where CD41 and Flk1 are co-expressed. **c** Visualization of lineage reconstruction for human preimplantation embryo using Monocle. The left and right panels, respectively, show the results of lineage reconstruction by Monocle without and with preprocessing the scRNA-seq data using DrImpute

We found that both Monocle and TSCAN had significantly improved performance on pseudotime inference on all three tested datasets if the scRNA-seq data were preprocessed by DrImpute, as supported by the significant increase of both POS and Kendall’s rank correlation score (Fig. 4a). Figure 4b showed single cells of mouse early mesodermal development data in 2D space using PCA without (left panel) and with (right panel) imputing the dropout events using DrImpute, and a pseudotime trajectory was constructed using TSCAN. Without imputation (left panel), the pseudotime trajectory started from E7.75 and ended at E7.75, which was not consistent with the known biological observations. In contrast, with imputation (right), the pseudotime trajectory started from E6.5 and ended at E7.75, and both POS and Kendall’s rank correlation score significantly increased (POS increased from 0.66 to 0.89, and Kendall’s rank correlation increased from 0.5 to 0.63).

As another example, Fig. 4c depicted single cells of human preimplantation embryo data in 2D space using independent component analysis (ICA), with the pseudotime trajectory inferred by Monocle. When imputing dropout events using DrImpute (right panel), not only did the trajectory start from E3 and end at E7, but the trajectory was also clearer in the sense that the E5, E6, and E7 stages were more easily separated compared to the trajectory inference results from non-imputed data (left panel). Consequently, the POS and Kendall’s rank correlation score were significantly increased (POS from 0.61 to 0.94; Kendall’s rank correlation from 0.44 to 0.77).

In summary, these results suggested that imputing dropout events using DrImpute also improved the performance of pseudotime inference using Monocle and TSCAN.

## Discussion

Dropout events and large cell-level variability are characteristic of scRNA-seq data, which are different from bulk RNA-seq data. However, many statistical tools derived for scRNA-seq data in cell type identification, visualization, and lineage reconstruction did not model for dropout events. Thus, we proposed a method for imputing dropout events considering cell-level correlation and systematically compared the performance without and with the imputation of dropout events. Our results on nine scRNA-seq datasets showed that imputing the dropout events using DrImpute significantly improved the performance of existing tools on cell type identification, visualization, and lineage reconstruction.

We would like to emphasize that DrImpute is the very first algorithm that sequentially utilizes dropout imputation with existing tools for more effective analysis. There are some statistical tools that model dropout events for specific purposes, such as BISCUIT, ZIFA, CIDR, scImpute and MAGIC. However, none of these suggest and compare the sequential use of dropout imputation and existing methods. We developed DrImpute to impute dropout events and demonstrated that the sequential use of dropout imputation employing DrImpute followed by the use of existing tools greatly improved the performance of the existing tools.

One of the limitations of DrImpute is that it considers only cell-level correlation using a simple hot deck approach. The gene-level correlation also exists, and more sophisticated modeling would improve the performance of the imputation. Most missing value imputation methods in bulk RNA-seq utilize gene-level correlation to impute missing values; for example, LLSimpute uses a local gene-level correlation structure to build local linear regression models to estimate missing values [46]. One may improve the performance of DrImpute by modeling both cell-level and gene-level correlation.

## Conclusions

The main goal of the current study was to de-noise the biological noise in scRNA-seq data by imputing dropout events. We developed DrImpute and proposed the sequential use of DrImpute on existing tools that do not address dropout events. The results suggested that DrImpute greatly improved many existing statistical tools (pcaReduce, SC3, PCA, t-SNE, Monocle, and TSCAN) that do not address the dropout events in three popular research areas in scRNA-seq—cell clustering, visualization, and lineage reconstruction. In addition, DrImpute combined with pcaReduce, SC3 or t-SNE/kms showed higher performance in cell clustering than CIDR, which was specifically designed for the cell clustering of scRNA-seq data. DrImpute combined with PCA or t-SNE also demonstrated higher performance in 2D visualization than did ZIFA, which was specifically designed for the dimensional reduction of scRNA-seq data considering dropout events. Moreover, DrImpute imputed dropout events better than scImpute and MAGIC, as we have shown that the performance of t-SNE increased greatly in regard to cell clustering and visualization with DrImpute compared to that with scImpute. In summary, DrImpute can serve as a very useful addition to the currently existing statistical tools for single cell RNA-seq analysis.

## Methods

### Data preprocessing

Seven scRNA-seq datasets (Pollen, Usoskin, Deng, Blakeley, Treutlein, Zheng and Hrvatin) were used for cell clustering and visualization. Three temporal scRNA-seq datasets (Deng, Scialdone, and Petropoulos) were used for lineage reconstruction. Genes that were expressed in fewer than 2 cells were removed. The raw read counts were normalized by size factor [47], followed by log transformations (*log*_10_(*X* + 1)). Table 1 summarized the nine datasets used in this study.

### Imputation strategy

Specifically, let *X* be a *n* by *p* log transformed gene expression matrix, where *n* is the number of rows (genes) and *p* is the number of columns (cells). The (*i*, *j*)th component of *X* is represented as *x*_*ij*_. Let *H* be the number of clustering configurations (e.g. combinations of distance metric and number of clusters), and *C*_1_, *C*_2_, …, *C*_*h*_ are each clustering results. Given that the clustering of *C*_*h*_ is a true hidden cell classification, the expected value of a dropout event can be obtained by averaging the entries in the given cell cluster:

*E*(*x*_*ij*_| *C*_*h*_) = *mean*(*x*_*ij*_ ∣ *x*_*ij*_ are in the same cell group in clustering *C*_*h*_).

This step was also schematized in Additional file 1: Figure S1. The *E*(*x*_*ij*_| *C*_*h*_) was computed for each clustering result *C*_1_, *C*_2_, …, *C*_*H*_, and the final imputation for the putative dropout events *x*_*ij*_, and *E*(*x*_*ij*_), was computed as a simple averaging:

$$ E\left({x}_{ij}\right)= mean\left(E\left({x}_{ij}|C\right)\right)=\frac{1}{H}\sum \limits_{h=1}^HE\left({x}_{ij}|{C}_h\right) $$

### Base clustering

For the default clustering of *C*_1_, *C*_2_, …, *C*_*H*_, we used an approach similar to that of SC3. We first created a similarity matrix among cells using Pearson and Spearman correlations. K-means clustering was performed on the first 5% of the principal components of the similarity matrix and the number of clusters ranged from 10 to 15. Thus, the default setting had a total of 12 clustering results (two distance construction methods (Pearson, Spearman) times six numbers of clusters (10 to 15) for k-means clustering). This default setting was used for all the data analysis in this manuscript except for the down-sampling cells for the Blakeley dataset, which only had 30 cells. Its sample size was too low to use a default range for the number of clusters, so in this case, we used a clustering group size of 6 to 10.

### Choices of number clusters and k-means initialization

We evaluated the robustness of imputation results on different choices of the number of clusters: *k* = 10 − 15 (default), *k* = 10 − 20, *k* = 10 − 25 and *k* = 10 − 30, as well as different random number seeds for k-means initialization. The robustness was quantitatively measured as Pearson’s correlation coefficient of imputed zero entries between any two conditions (choices of *k* ranges and random seeds). We found that on two tested datasets (Pollen and Usoskin), the imputed results were generally robust on different choices of *k* ranges and random seeds (Additional file 1: Figure S4a and b). We therefore chose *k* = 10 − 15 as the default parameters since it needed less running time and would be more efficient for processing large-scale datasets.

Moreover, our experience with DrImpute suggested that the range of *k* needed to be no less than the *real* number of cell clusters (though unlike other methods such as scImpute, pcaReduce or SIMLR, the exact number of expected clusters do not need to be specified) [23, 25, 48]. For heterogeneous scRNA-seq datasets where more than 10 cell clusters are expected, a higher range of *k* may be necessary to obtain most accurate imputation results.

### Imputing large-scale scRNA-seq datasets

In order for DrImpute efficiently imputing large-scale scRNA-seq datasets, we have improved the running efficiency in two ways. First, for large scRNA-seq datasets, we adopted a sampling-based algorithm without computing the full cell-cell distance matrices [49]. Second, in order to speed up k-means for very large scRNA-seq datasets, we have implemented a mini-batch k-means [50]. Both sampling-based PCA of distance matrix and mini-batch k-means could be performed in parallel and therefore greatly improve the running time of DrImpute. It took on average 750 s for DrImpute imputing a scRNA-seq datasets with 10,000 cells (Additional file 1: Figure S6). It should be noted that for large-scale sparse scRNA-seq datasets, DrImpute imputed significantly less zero entries in the gene-cell expression matrix (Additional file 1: Figure S3).

### Software implementations and applications

The pcaReduce software was downloaded from the authors’ GitHub (https://github.com/JustinaZ/pcaReduce). We performed the analysis using the S and M options with the default setting.

The SC3 package was downloaded from R Bioconductor (http://bioconductor.org/packages/release/bioc/html/SC3.html). To ensure the consistency of the comparison with other tools, the gene filtering option was turned off (gene.filter = FALSE). Other options were set as default.

The Rtsne package and kmeans function in R program were used for t-SNE (perplexity = 9) followed by k-means. The log transformed expression data were centered as the gene level. We used the R kmeans function with the option iter.max = 1e + 09 and nstart = 1000 for stable results.

ZIFA software was then downloaded (https://github.com/epierson9/ZIFA). We used block_ZIFA with k = 15 for all data analysis, and we used the first two dimensions for visualization and evaluation.

Monocle was downloaded from the R Bioconductor page (https://bioconductor.org/packages/release/bioc/html/monocle.html). In Monocle analysis, we first selected genes expressed in at least 50 cells and then selected differentially expressed genes using the differentialGeneTest() function (qval < 0.01). If there were no differentially expressed genes using the provided test, all genes expressing at least 50 cells were used for the subsequent analysis.

TSCAN was downloaded from the R Bioconductor page (https://www.bioconductor.org/packages/release/bioc/html/TSCAN.html). All default settings were used for TSCAN.

The MAGIC package was downloaded from GitHub (https://github.com/KrishnaswamyLab/magic), and the R version of MAGIC was used for the analysis. As suggested in their manual page, we used settings *t* = 6 and rescale_percent = 0.99.

The scImpute package was downloaded from GitHub (https://github.com/Vivianstats/scImpute). We used the settings drop_three = 0.5 and four CPU cores for the analysis. The Kcluster parameter was set as the expected number of cell clusters in each dataset (e.g. Kcluster = 11 for Pollen dataset).

### Evaluating the robustness of cell clustering

To evaluate the robustness of various cell clustering methods (Additional file 1: Figure S5a and b), we down-sampled 100 genes (or about one-third of the cells) at random. PcaReduce, SC3, and t-SNE followed by k-means were applied to the down-sampled datasets, with or without imputing the dropout events using DrImpute and CIDR. The above processes were repeated 100 times. The mean pairwise ARI of the clustering results from a total of 100 × 99/2 pairs of repeated runs was used as a robustness criterion using down-sampled genes (or cells). Note that when the cells were down-sampled, the overlapped cells were used for computing partial ARIs.

## Additional file

Additional file 1: Supplementary Information. A pdf file that contains additional figures and figure legends omitted from the main paper. (PDF 1217 kb)

## Abbreviations

ARI: Adjusted Rand Index
bulk RNA-seq: Bulk RNA sequencing
CIDR: Clustering through Imputation and Dimensionality Reduction
ICA: Independent component analysis
NF: neurofilament containing
NP: peptidergic nociceptors
PCA: Principal component analysis
PEP: peptidergic nociceptors
POS: Pseudo-temporal Ordering Score
scRNA-seq: single cell RNA sequencing
TH: tyrosine hydroxylase containing
t-SNE: t-distributed stochastic neighbor embedding
ZIFA: Zero Inflated Factor Analysis
