# Supplementary material for: DrImpute: imputing dropout events in single cell RNA sequencing data
Source: BMC Bioinformatics. 2018 Jun 8;19:220. doi: 10.1186/s12859-018-2226-y (PMC5994079; doi:10.1186/s12859-018-2226-y)
Supplement: Supplementary file 1 — Supplementary Information. A pdf file that contains additional figures and figure legends omitted from the main paper. (PDF 1217 kb) [file 12859_2018_2226_MOESM1_ESM.pdf]

Supplementary Figure 1

Cells

Genes

C1	C2	C3	C4	C5	C6	C7	C8	C9	C10
2	3	3	0	2	0	0	0	0	0
3	3	0	4	0	0	0	0	0	1
0	1	0	0	0	5	0	6	3	6
3	2	2	2	2	0	0	0	1	0

Cell Clustering

C1, C2, C3
C4, C5

C6, C7, C8
C9, C10

Imputation

Genes

C1	C2	C3	C4	C5	C6	C7	C8	C9	C10
2	3	3	2	2	0	0	0	0	0
3	3	2	4	2	0.2	0.2	0.2	0.2	1
0.2	1	0.2	0.2	0.2	5	4	6	3	6
3	3	2	2	2	0.2	0.2	0.2	1	0.2

Supplementary Figure 1. Basic procedure for clustering-based imputation. Upper matrix is a gene by cell matrix. After clustering on gene by cell matrix, we observe C1–C5 as one cluster and C6–C10 as the other cluster. Imputation is performed by averaging each cluster.

Supplementary Figure 2

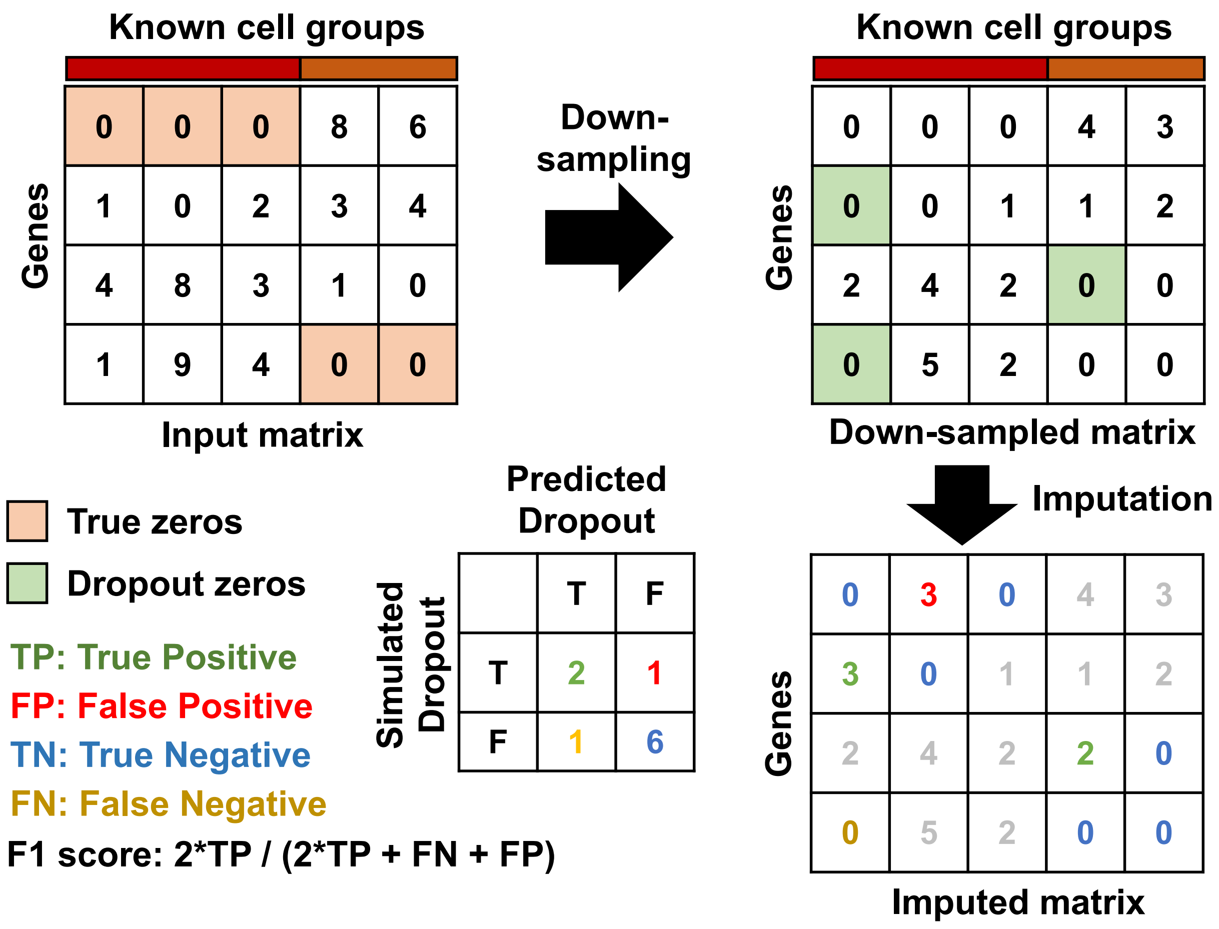

Supplementary Figure 2. Overview of the down-sampling studies on discriminating true zeros and dropout zeros. We defined the *true zeros* as the genes where expression levels are consistently zero across all cells belonging to one cell cluster. To generate the *dropout zero*, we randomly down-sampled the raw sequencing reads to a certain percent (e.g. 25%) of the total number of reads, mapped the sampled reads onto the genome and computed the corresponding gene-cell read count matrices. We defined *dropout zero* as the genes where expression levels are zero in the down-sampled datasets, but are positive in the full dataset. The imputed zero events could be therefore grouped into four situations: (1) true positive (TP, imputed dropout zeros), (2) true negative (TN, non-imputed true zeros), (3) false positive (FP, imputed true zeros) and (4) false negative (FN, non-imputed dropout zeros). The F1 score (the harmonic mean of precision and recall) was used to evaluate the imputation performance of each method on down-sampled datasets.

Supplementary Figure 3

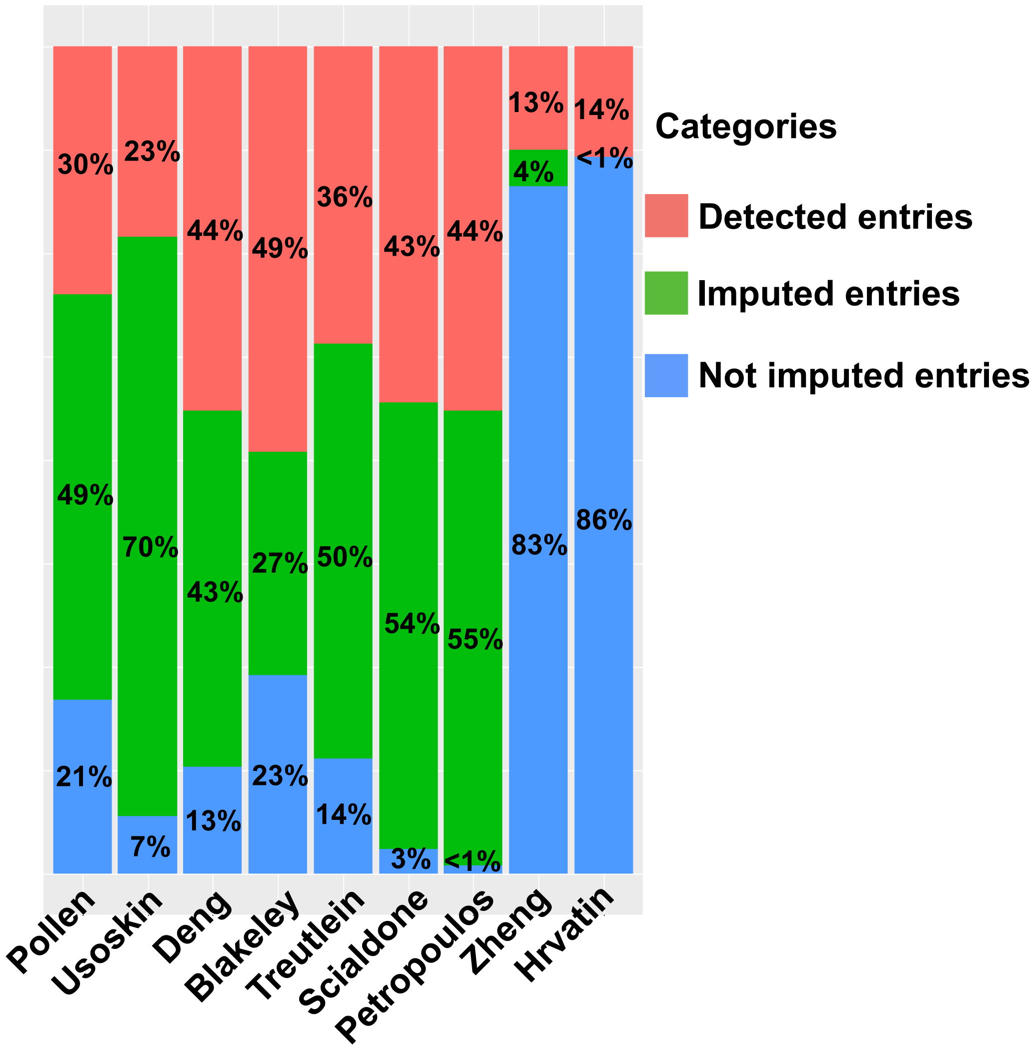

Supplementary Figure 3. Overview of percent of detected, imputed and not imputed entries in scRNA-seq datasets used in this study. Percentage of detected (input read count > 0), imputed (input read count is zero and the imputed read count is positive), and not imputed entries (both input and imputed read count are zeros) for nine different scRNA-seq datasets. Genes that were expressed in less than 2 cells were excluded before this analysis.

Supplementary Figure 4

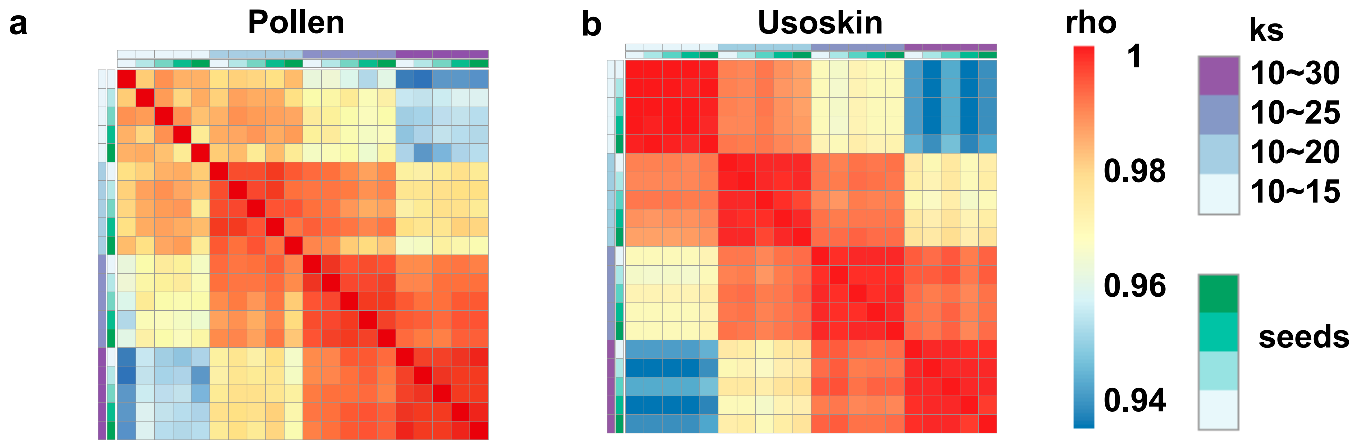

Supplementary Figure 4. DrImpute was robust on the different choices of the k ranges and random seeds for the (a) Pollen and (b) Usoskin datasets. The robustness of imputation results were evaluated on different choices of number clusters: $k = 10 - 15$ (default), $k = 10 - 20$, $k = 10 - 25$ and $k = 10 - 30$, as well as different random number seeds (1 - 5) for k-means initialization. The robustness was quantitatively measured as Pearson's correlation coefficient of imputed zero entries between any two conditions (choices of k ranges and random seeds). The color of the heatmap indicates the Pearson's correlation coefficient.

Supplementary Figure 5

a

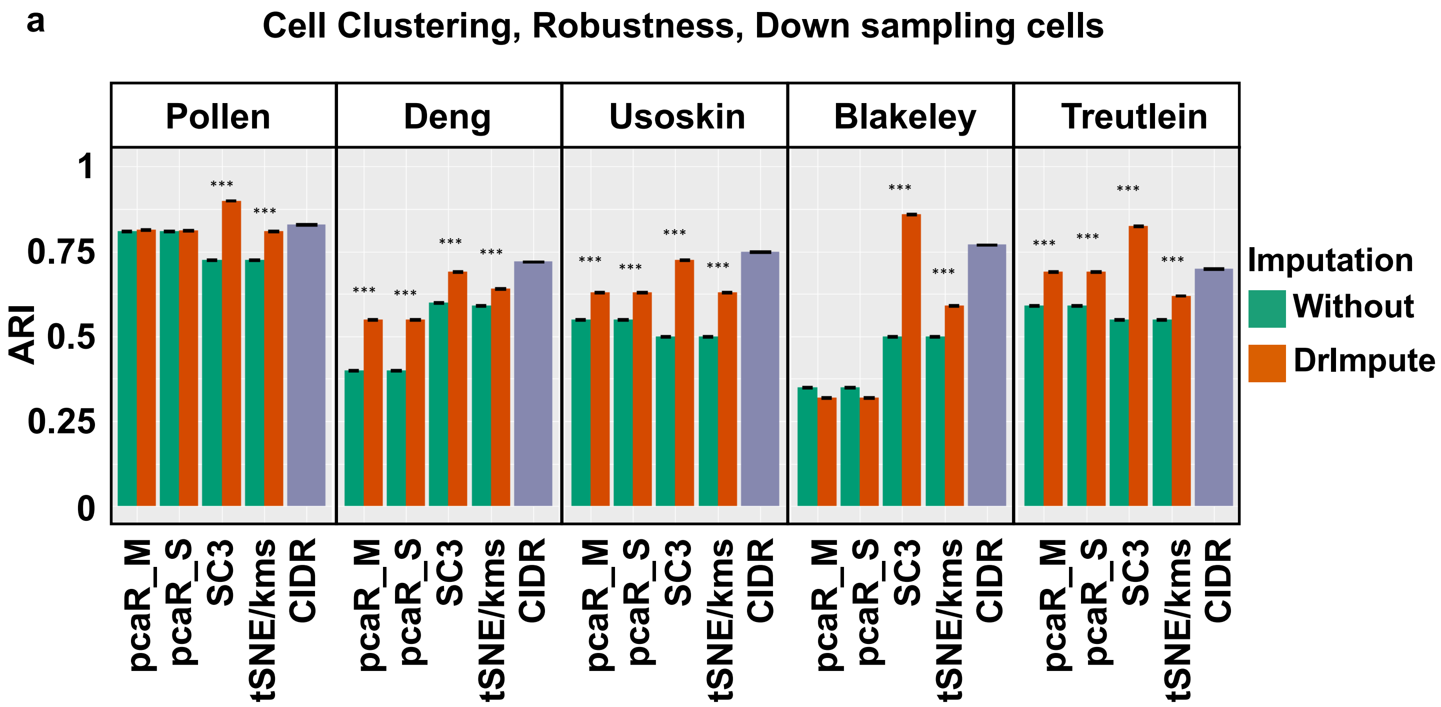

b

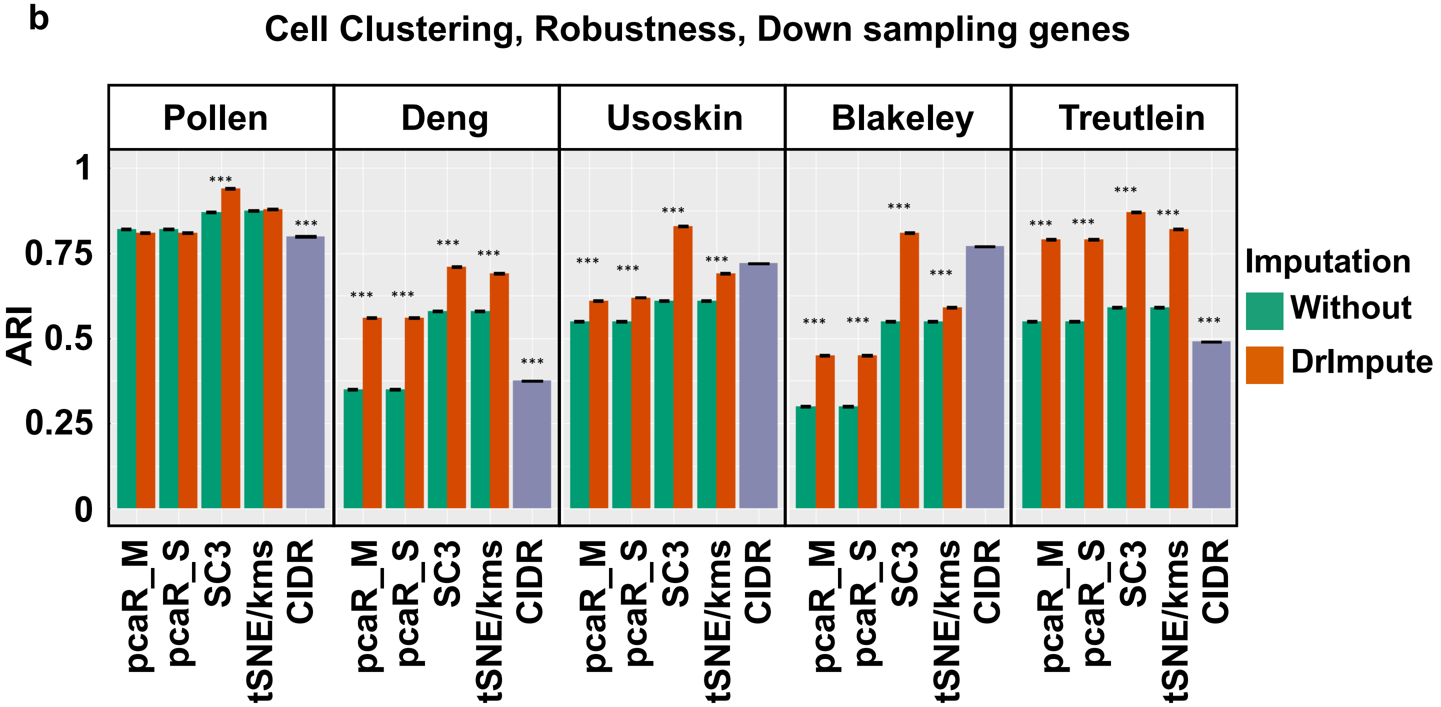

Supplementary Figure 5. DrImpute significantly improved the performance of the existing tools for cell type identification in robustness criteria. To account for robustness, original datasets were down-sampled by cells (a) or by genes (b); we recorded clustering results for each data subset. ARIs are calculated for each pair of data subsets. Barplot represents averaged ARIs. Blue interval represents one plus or minus standard deviation of the data. Black interval represents one plus or minus standard error of the data. Wilcoxon rank sum test is performed to compare before and after imputation. For down-sampled cells, 16 out of 20 cases are improved. For down-sampled genes, 18 out of 20 cases are improved ($*** \ p \ \mathrm{value} < 0.001$).

Supplementary Figure 6

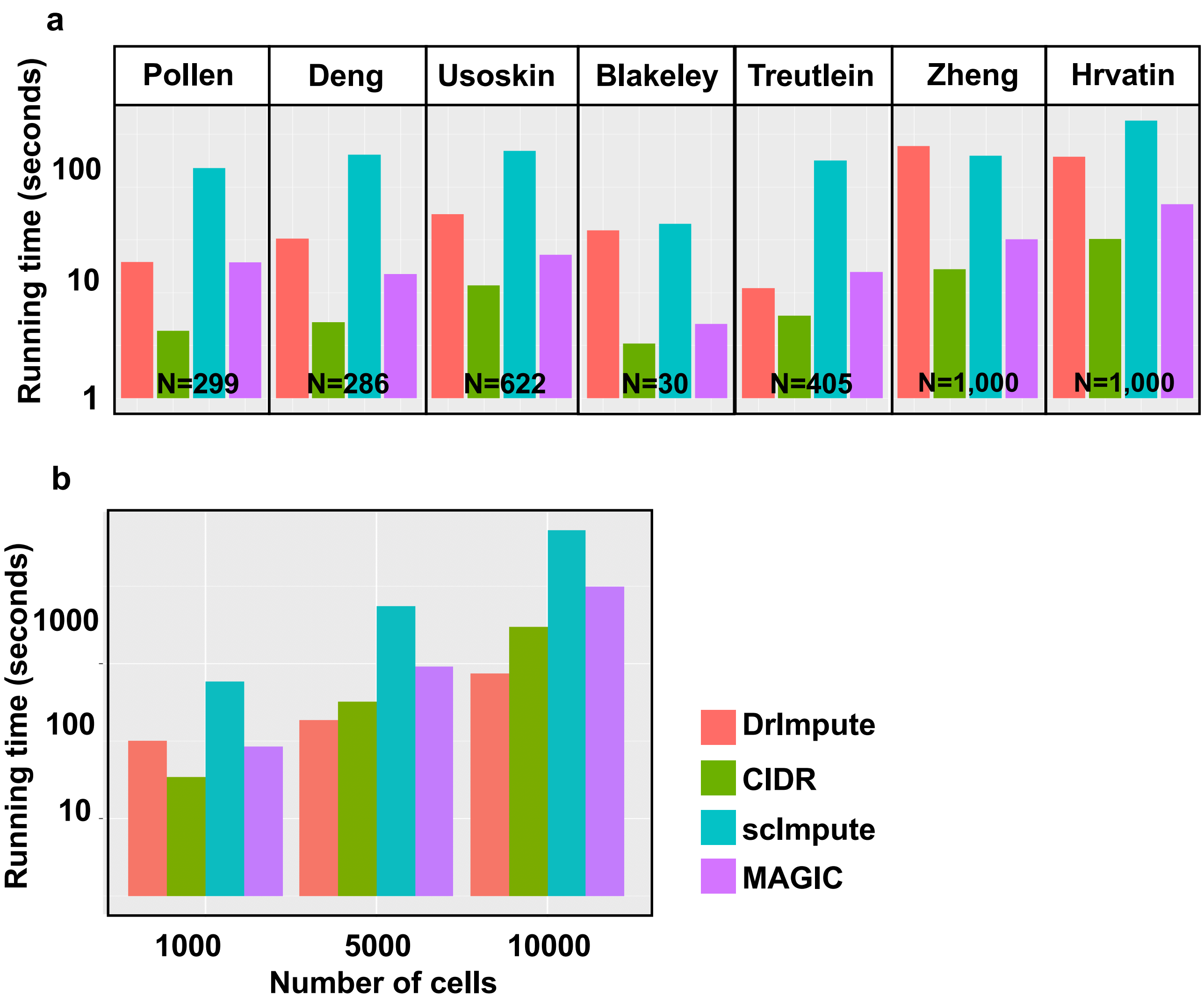

Supplementary Figure 6. DrImpute is efficient on imputing large-scale scRNA-seq datasets. (a) The running time of DrImpute, CIDR, scImpute and MAGIC on nine tested datasets are presented. The y-axis indicates the running time in seconds. **(b)** The running time of DrImpute, CIDR, scImpute and MAGIC on randomly sampled 1,000, 5,000 and 10,000 cells from the Zheng dataset are presented. All the analysis were performed on Intel Xeon 2.4GHz CPU. For both DrImpute and scImpute, 4 CPU cores were used for the analysis.
